# Presleep vs. Daytime Consumption of Casein-Enriched Milk: Effects on Muscle Function and Metabolic Health After Sleeve Gastrectomy

**DOI:** 10.3390/nu17172750

**Published:** 2025-08-25

**Authors:** Nida Yıldız, Halil Coşkun, Mert Tanal, Murat Baş, Duygu Sağlam

**Affiliations:** 1 Department of Nutrition and Dietetics, Institute of Health Sciences, Acıbadem Mehmet Ali Aydınlar University, 34752 Istanbul, Turkey; nida.yildiz@memorial.com.tr; 2 Obesity and Metabolic Surgery Center, Memorial Şişli Hospital, 34385 Istanbul, Turkey; merttanal@yahoo.com; 3 BariatrikLab Obesity and Metabolic Surgery Center, 34379 Istanbul, Turkey; halilcoskun@hotmail.com; 4 Department of Nutrition and Dietetics, Faculty of Health Sciences, Acıbadem Mehmet Ali Aydınlar University, 34752 Istanbul, Turkey; murat.bas@acibadem.edu.tr

**Keywords:** casein, muscle function, nutritional timing, sleeve gastrectomy, bariatric surgery, protein intake, body composition

## Abstract

**Background/Objectives**: This randomized controlled trial aimed to evaluate the effects of casein-enriched milk (CEM) consumption and its timing (presleep vs. during the day) in the early postoperative period on body composition, muscle strength, physical function, and biochemical parameters in individuals undergoing laparoscopic sleeve gastrectomy (SG). **Methods:** Forty-five adults (60% female, 40% male; mean age 35.1 ± 9.7 years; mean BMI 41.4 ± 4.9 kg/m^2^) undergoing SG were randomly assigned to three groups: (1) 15 g protein CEM (12 g casein) presleep, (2) the same CEM during the day, or (3) standard-protein diet without supplementation. The primary endpoint was change in fat-free mass (FFM) at 12 weeks; secondary endpoints included handgrip strength, 30 s sit-to-stand test, and serum total protein, albumin, and prealbumin. Assessments were performed preoperatively and at weeks 4, 8, and 12. **Results:** No significant differences were found between the groups in terms of body composition, muscle strength, or physical performance measurements (*p* > 0.05). However, a significant increase in handgrip strength was observed over time in Groups 1 and 2 (*p* < 0.05), which was not observed in Group 3. Prealbumin levels at week 12 were 0.3 ± 0.0 mg/dL in Group 1 and 0.2 ± 0.0 mg/dL in Group 2, both higher than 0.2 ± 0.0 mg/dL in Group 3 (*p* < 0.05). No significant differences were found in albumin and total protein levels (*p* > 0.05). **Conclusions:** Early postoperative CEM consumption following SG did not significantly affect body composition or physical performance; however, the higher prealbumin levels indicate that this marker may be more sensitive in detecting early protein response, highlighting its potential clinical relevance in monitoring nutritional status after bariatric surgery.

## 1. Introduction

Obesity is a chronic disease characterized by abnormal or excessive fat accumulation in the body [1]. According to the World Health Organization, in 2022, 2.5 billion adults were overweight and 890 million were obese, representing 43% and 16% of the global adult population, respectively [2]. Since 1990, the prevalence of obesity has more than doubled, underscoring its role as a major global public health issue [3,4].

In cases of severe obesity, bariatric surgery has emerged as one of the most effective treatment methods, achieving sustainable weight loss and remission of metabolic diseases [5,6]. Sleeve gastrectomy (SG), the most frequently performed procedure, induces durable body weight reduction by restricting food intake through the resection of a large portion of the stomach [7].

However, postoperative weight loss is not solely derived from fat mass but also from fat-free mass (FFM) [8]. The reduction in FFM may lead to declines in muscle strength and physical capacity [9]. Although improvements in physical function have been reported postoperatively, it remains unclear whether these improvements result directly from the surgery itself or from increased physical activity [10,11]. In addition, excessive FFM loss may contribute to a decrease in basal metabolic rate and subsequent metabolic adaptation [12]. Maintaining muscle mass is crucial for functional recovery and metabolic stability after bariatric procedures. Therefore, understanding how the quantity and timing of protein intake affect this process is highly relevant from a clinical perspective [13].

The loss of FFM is closely linked to insufficient protein intake [9]. Current bariatric surgery guidelines recommend a minimum protein intake of at least 60 g/day and 1.5 g/kg of ideal body weight per day [14]. Adequate protein intake is essential not only for preserving muscle mass but also in supporting wound healing, immune function, and overall recovery in the postoperative period [15]. Beyond the absolute quantity of protein consumed, recent evidence suggests that the timing of protein intake may also play an important role in modulating muscle mass preservation and metabolic outcomes [16]. Despite clear recommendations on total protein intake, there is limited evidence on how the timing of protein ingestion—particularly the use of slow-digesting proteins like casein—might influence recovery outcomes following SG.

Casein, a slow-digesting milk protein, is considered ideal for presleep supplementation due to its ability to sustain amino acid release and thereby potentially enhance overnight muscle protein synthesis [17]. Randomized controlled studies in young, healthy men engaged in resistance exercise demonstrated that consuming casein presleep increased whole-body protein synthesis rates throughout the night and improved net protein balance [18]. Similarly, another study reported that presleep casein intake enhanced myofibrillar protein synthesis rates [19]. While these findings highlight casein as an optimal protein source for presleep supplementation [16], the majority of evidence is derived from healthy or athletic populations, and its relevance to bariatric patients remains uncertain.

Alongside functional and body composition outcomes, biochemical markers provide valuable tools for assessing early postoperative nutritional recovery. Prealbumin, in particular, is widely recognized as a sensitive and early biomarker of protein status. With a short half-life of approximately 2–3 days, prealbumin rapidly reflects short-term changes in protein intake and utilization [20]. This dynamic responsiveness makes prealbumin clinically useful for monitoring early nutritional recovery, especially in contexts such as bariatric surgery where protein intake may be insufficient [21]. Incorporating prealbumin as a biochemical marker provides additional insight into the relationship between protein timing and early nutritional status after SG.

Despite the clinical importance of both muscle preservation and nutritional monitoring, studies evaluating the impact of protein timing in the postoperative bariatric population remain limited. This study aims to address this gap by evaluating the impact of the timing of casein-enriched milk consumption (presleep vs. during the day) on body composition, muscle function, and initial biochemical indicators of nutritional status (prealbumin, albumin, and total protein) in individuals recovering from SG.

## 2. Materials and Methods

### 2.1. Study Design and Ethical Approval

This study is a randomized controlled trial conducted between October 2023 and October 2024 at the Obesity and Metabolic Surgery Center of Memorial Şişli Hospital with patients who underwent laparoscopic SG and were under regular dietitian follow-up for at least 12 weeks post-surgery.

A total of 57 individuals were enrolled. Of these, 12 participants (21.1%) did not complete the study: four (33.3%) due to failure to attend scheduled follow-up visits and eight (66.7%) due to non-adherence to the dietary intervention or incomplete dietary records. Analyses were therefore conducted on the remaining 45 participants.

The sample size was determined through power analysis by using the GPower 3.1.9.2. (Heinrich-Heine- Universitat Düsseldorf, Düsseldorf, Germany) software. To obtain statistical power with an alpha (type I error) of 0.05 and 1-beta (power) of 0.8, effect sizes (Cohen’s d: 0.5) were determined. Participants were randomly assigned to three groups (presleep consumption, during the day consumption, and control) in equal numbers (*n* = 15 per group) using a computer-generated simple randomization method. This was a non-blinded study; neither participants, clinicians, nor outcome assessors were blinded to group allocation.

**Figure 1:**
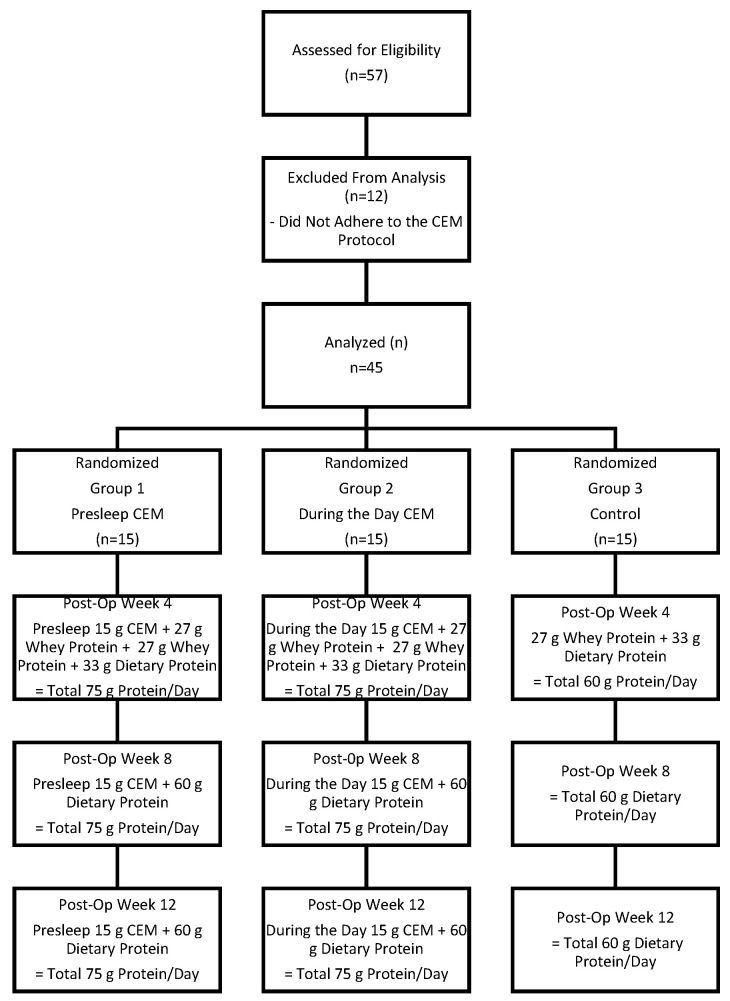
Flowchart of the study groups according to the postoperative CEM supplementation schedule. CEM: Casein-Enriched Milk.

The study protocol was approved by the Acıbadem University and Acıbadem Healthcare Institutions Medical Research Ethics Committee (decision number 2023-15/530). Written informed consent was obtained from all participants prior to inclusion. Anthropometric measurements, body composition, muscle strength, dietary intake records, physical function tests, and biochemical assessments were collected preoperatively and at 4, 8, and 12 weeks postoperatively (Figure 1).

### 2.2. Setting and Participants

Inclusion criteria were as follows: participants aged 18–65 years with a Body Mass Index (BMI) ≥ 40 kg/m^2^, or a BMI ≥ 35 kg/m^2^ with at least one obesity-related comorbidity (e.g., type 2 diabetes, hypertension, sleep apnea).

Exclusion criteria were undergoing any obesity or metabolic surgery other than SG; having a BMI < 35 kg/m^2^; a history of cow’s milk allergy; or alcohol or substance utilize.

### 2.3. Nutritional Intervention

Participants were randomized into three groups.

Group 1 (Presleep): In addition to a standard diet containing 60 g/day of protein, participants consumed 15 g of protein (12 g casein, 3 g whey protein) in milk presleep, resulting in a total daily protein intake of 75 g.

Group 2 (During the day): Participants received the same standard diet supplemented with 15 g of protein (12 g casein, 3 g whey protein) in milk consumed during the day, for a total daily protein intake of 75 g.

Group 3 (Control): Participants received only the standard diet containing 60 g/day of protein.

Casein-Enriched Milk (CEM): CEM (Ak Gıda, Sakarya, Turkey) was utilized as the intervention product. Each serving provided 125 kcal, with 48% of the energy from protein, 43% from carbohydrates, and 9% from fat. Each portion contained 15 g of protein (12 g casein and 3 g whey).

All patients were given one packet of Barivital (Pharmago Health Products and Foreign Trade Inc., Istanbul, Turkey) protein supplement daily for the first four weeks. One packet of this protein powder contains 187.61 kcal of energy, with 58.64% of the energy coming from protein, 25.07% from carbohydrates, and 11.13% from fat. Each packet contains 27 g of whey protein (95% concentrate, 5% isolate).

Adherence to the nutritional intervention was monitored through daily dietary records completed by each participant, documenting the timing and amount of CEM consumed. These records were reviewed by the dietitian follow-up sessions.

### 2.4. Anthropometric Measurements and Body Composition

Height was measured using the ADE M320600-01 stadiometer (ADE GmbH, Hamburg, Germany). Body weight and height were utilized to calculate Body Mass Index (BMI, kg/m^2^). The following formulas were applied:

Body Mass Index (BMI) = Weight (kg)/height^2^ (m^2^)

%Excess Weight Loss (%EWL) = [(Preoperative weight − current weight)/(preoperative weight − ideal weight)] × 100

The Excess Body Mass Index Loss (%EBMIL) was calculated using the following formula: [(Preoperative BMI − current BMI)/(preoperative BMI − 25)] × 100 [22].

Body composition, including body fat percentage, fat mass, and FFM, was assessed using a segmental body composition analyzer (Tanita MC-780S/ST; Tanita Corp., Tokyo, Japan). Standardized pre-test conditions were applied prior to bioelectrical impedance analysis: participants refrained from exercise for at least 8 h, fasted for 6–8 h (including water), and removed all metal objects that could interfere with the measurement [23].

### 2.5. Handgrip Strength

Handgrip strength was assessed using a Takei T.K.K.5401 GRIP-D dynamometer (Takei Scientific Instruments Co., Ltd., Tokyo, Japan). Measurements were performed three times consecutively without rest intervals. For each trial, participants were instructed to squeeze the dynamometer maximally for 3 s, and the mean value of the three attempts was recorded [24].

### 2.6. Physical Function

The 30 s sit-to-stand test was utilized to assess lower extremity strength. The test was performed according to standardized protocol: participants were seated on a chair without armrests at a fixed seat height, with the back straight, arms crossed over the chest, and feet flat on the ground. Following the examiner’s command, participants repeatedly stood up and sat down for 30 s. This test provides a reliable and practical measure of both muscular strength and endurance [25].

### 2.7. Biochemical Tests

Blood samples were collected after an overnight fast for the assessment of protein status, including total protein, albumin, and prealbumin levels. Serum albumin levels were measured by the bromocresol green colorimetric method, and total protein levels were determined by the biuret colorimetric method, both performed on the Abbott Alinity c automated analyzer (Abbott Laboratories, Chicago, IL, USA) using the manufacturer’s commercial kits (Abbott). Serum prealbumin levels were measured by immunonephelometry on a Siemens BN ProSpec nephelometer (Siemens Healthineers, Erlangen, Germany) using commercial reagents from Siemens Dade Behring, according to the manufacturer’s instructions. Reference ranges were defined as follows: total protein 6.0–8.0 g/dL, albumin 3.5–5.0 g/dL, and prealbumin 0.16–0.30 g/L.

### 2.8. Dietary Intake and Nutritional Counseling

Dietary intake was assessed four times in total, before bariatric surgery and at the end of the 4th, 8th, and 12th weeks post-surgery, using the 24 h recall method. Data were analyzed using the NUTRISURVEY software (version 5.0; University of Hohenheim, Stuttgart, Germany).

Nutritional counseling was provided to all patients participating in the study by a registered dietitian. Patients were advised to start with a low-sugar clear liquid diet postoperatively, gradually progressing to clear liquids, then to pureed or soft foods, and finally to solid, chewable foods after 4 weeks [26].

### 2.9. Statistical Analysis

The findings obtained in the study were evaluated using SPSS version 26.0 for Windows (SPSS; Chicago, IL, USA) statistical package program for statistical analysis. Demographic characteristics of the participants were examined descriptively. Before comparing quantitative data, the normality of the variables was tested using the Shapiro–Wilk method. Non-parametric tests (Kruskal–Wallis test, Friedman test) were applied to values that did not follow a normal distribution based on the Shapiro–Wilk test values. On the other hand, parametric tests (One-Way ANOVA test, Repeated Measures) were utilized for values that were suitable for normal distribution. The results obtained from the comparisons were evaluated at a 95% confidence interval; *p* < 0.05 significance level.

## 3. Results

A total of 57 patients were initially included in the study. However, 12 patients were excluded due to failure to consume the casein-enriched milk product as recommended and non-compliance with the protocol. While compliance-related exclusions were accounted for, the potential influence of varying adherence levels on outcomes remains a consideration. Analyses were conducted on 45 patients randomly assigned to three groups (*n* = 15). The groups were designated as Group 1 (presleep), Group 2 (during the day), and Group 3 (control). Sixty percent of the participants were female, and 40% were male, with a mean age of 35.1 ± 9.7 years.

The preoperative anthropometric measurements of the patients participating in the study were compared across groups. The patients’ BMI (kg/m^2^), FFM (kg), FFM percentage (%), Skeletal Muscle Mass (SMM), (kg), SMM percentage (%), Body Fat Mass (BFM) (kg), BFM percentage (%), Total Body Water (TBW) (kg), and TBW percentage (%) were found to demonstrate no statistically significant differences between groups (*p* > 0.05). The preoperative anthropometric measurements of the patients are presented in Supplementary Table S1.

There were no statistically significant differences between groups in patients’ EWL, EBMIL, FFM, SMM, BFM, and TBW at 4, 8, and 12 weeks post-op (*p* > 0.05) (Table 1). As shown in Table 1, mean SMM was more stable in Groups 1 and 2 than in the control group, with Group 1 showing the smallest reduction over the 12-week period. Although not statistically significant, Group 2 exhibited the smallest numerical reduction in FFM over 12 weeks, followed by Group 1, while Group 3 showed the greatest reduction. In terms of SMM, Group 1 demonstrated the least decrease, followed by Group 2, with the largest loss observed in Group 3. For BFM, Group 1 achieved the greatest numerical reduction, closely followed by Group 3, whereas Group 2 showed the smallest decrease. Although the differences did not reach statistical significance, the observed trends in Group 1 suggest the potential benefits of presleep casein supplementation.

There was no statistically significant difference between the groups in handgrip strength and the 30 s sit-to-stand test (*p* > 0.05) (Table 2). As shown in Table 2, improvements in both right and left handgrip strength were more pronounced in Groups 1 and 2 than in Group 3. From baseline to week 12, all groups showed improvements in right and left handgrip strength and in the sit-to-stand test. The greatest increase in right handgrip strength was observed in Group 1, followed by Group 2, with the smallest increase in Group 3. A similar pattern was seen for left handgrip strength. For the sit-to-stand test, the highest improvement occurred in Group 1, followed by Group 2, and the lowest improvement in Group 3. However, when comparing the groups over time, Group 1 and Group 2 demonstrated statistically significant differences in handgrip strength and the 30 s sit-to-stand test, a physical function test, over time (*p* < 0.05). Group 3 demonstrated no statistically significant difference in handgrip strength (*p* > 0.05) however a statistically significant difference in the physical function test (*p* < 0.05). Detailed handgrip strength and physical function test results are presented in Supplementary Table S2.

Prealbumin and albumin levels were significantly different between groups in the preoperative period (*p* < 0.05). Prealbumin and albumin levels were higher in Group 1 compared to Group 2. Significant differences in prealbumin levels were also observed between groups at postoperative weeks 4, 8, and 12 (*p* < 0.05), with median values in Groups 1 and 2 being higher than those in Group 3. As shown in Table 3, albumin and total protein levels remained relatively stable across all groups, with only minor fluctuations from baseline values. Although not statistically significant, albumin and total protein levels remained close to preoperative values across all groups throughout the 12-week follow-up, showing only minor fluctuations and an overall stable trend. However, total protein and albumin levels did not demonstrate significant differences between groups throughout the preoperative and postoperative periods (*p* > 0.05) (Table 3).

## 4. Discussion

This study is one of the first to evaluate the effects of CEM timing (presleep or during the day) on body composition, muscle strength, physical performance, and certain biochemical markers in individuals undergoing SG. Comparing the intervention groups with a group that only received dietary protein allowed for a clearer assessment of the potential effects of casein intake timing.

A significant decrease in FFM is observed after bariatric surgery [9]. Martinez et al. [27] reported a significant decrease in FFM at week 4 post-SG. In our study, a decrease in FFM was observed in all groups at postoperative weeks 4, 8, and 12. However, despite the consumption of CEM at presleep or during the day, no statistically significant difference in FFM was determined between the intervention groups and Group 3. This finding suggests that the timing of protein supplementation in the early post-bariatric surgery period may not provide a significant advantage in FFM.

Schollenberger et al. [28] reported that FFM loss was lower in the group receiving protein supplementation; however, this difference was only significant in individuals who utilized the supplementation regularly.

In the early postoperative period, the use of a very low-protein diet may lead to more pronounced deterioration in body composition [29]. Considering that all individuals in our study were fed at or above the daily minimum protein level recommended by the American Society for Metabolic and Bariatric Surgery (≥ 60 g/day) [14], it can be inferred that the total protein intake amount, independent of timing, may have a more decisive effect on FFM. Additionally, López-Gómez et al. [29] emphasized that low-protein diets may cause more pronounced impairments in body composition during the postoperative period. Our findings are generally consistent with the literature; however, the reasons for the limited effect of timing should be explored more comprehensively through other clinical and functional parameters.

In our study, when handgrip strength was assessed in the post-bariatric surgery period, an increase in strength over time was observed in Group 1 and Group 2, which consumed CEM; however, no significant change was detected in Group 3, which did not consume this product. Although no statistically significant differences were found between the groups, the trend observed only in the groups consuming CEM suggests that the product may have a potential effect on functional muscle strength. The similar increases observed in both the night and day consumption groups support the notion that this effect may be more related to the product’s composition than to timing. Indeed, the literature indicates that casein intake before sleep may increase muscle protein synthesis rates, thereby providing positive effects on muscle tissue, particularly during the night [30]. Given casein’s slow digestion and sustained amino acid release, it has been hypothesized that it supports nocturnal muscle protein synthesis. However, our findings suggest that this potential benefit may not be realized during the acute postoperative period, possibly due to competing catabolic processes or altered gastrointestinal physiology post-SG. A meta-analysis by Jung et al. [31] also reported no significant change in handgrip strength after SG and gastric bypass surgery, although increases were observed in these individuals. These findings are consistent with previous meta-analyses reporting that muscle strength can be maintained or improved despite muscle mass loss following bariatric surgery. Although no significant difference in handgrip strength was observed between groups, the increasing trend observed in groups consuming casein-containing products is noteworthy. In this context, the sit-to-stand test (STS) was also applied in our study to evaluate muscle function more comprehensively.

The STS test revealed that physical function improved over time following surgery. However, no statistically significant differences were found between groups in terms of the magnitude of improvement in performance. This result suggests that the increase in physical function after bariatric surgery likely occurred spontaneously due to the effects of surgery (weight loss, reduced inflammation, lifestyle changes, etc.). Improvements in STS performance may have resulted from reductions in joint stress induced by surgery, improved cardiometabolic status, or increased daily activity levels, independently of protein supplementation. Additionally, it was understood that the timing of protein supplementation (presleep vs. during the day) did not provide an additional contribution to this process. This result is supported by similar studies in the literature: In a randomized controlled trial conducted by Hirsch et al. [32], a protein supplement group and a daily ready-to-drink protein shake group were compared with a standard-of-care group for 12 weeks after bariatric surgery. STS performance improved significantly in both groups; however, the difference between the groups was not significant. Oliveira et al. [9] compared the effects of a resistance exercise program with and without protein supplementation; they reported that STS and muscle function improved similarly with exercise; however, protein supplementation did not contribute significantly to this improvement. Similarly, in a randomized controlled trial conducted by Oppert et al. [33], resistance exercise administered post-bariatric surgery significantly improved STS performance; however, additional protein supplementation did not contribute statistically significantly to this improvement. Based on these findings, it is understood that protein supplements do not create a significant difference in physical function outcomes, and therefore, protein timing alone does not contribute to functional gains following bariatric surgery.

Protein deficiency is frequently monitored in clinical practice by serum protein levels such as albumin and prealbumin [34]. Prealbumin, which has a shorter biological half-life than albumin, is considered a more sensitive marker because it reflects acute changes in nutritional status more rapidly [35]. Baseline differences in albumin and prealbumin may reflect random variation despite randomization. In our study, prealbumin levels were found to be higher in Groups 1 and 2, compared to Group 3 after surgery. However, no significant differences were observed between the groups in terms of albumin and total protein levels. This suggests that casein-based protein supplementation may have short-term positive effects on visceral protein synthesis; however, it does not cause significant changes in markers such as albumin, which have a delayed response. Erstad et al. [36] reported that while albumin levels did not change significantly after parenteral nutrition in surgical patients, prealbumin levels increased statistically significantly. Wang et al. [37] demonstrated that high protein intake (1.5 g/kg/day) in the early period significantly increased prealbumin levels in intensive care patients. The increase observed in prealbumin levels in our study is consistent with this literature. However, studies evaluating the effect of protein supplementation on prealbumin levels in the post-bariatric surgery period are quite limited. In our study, presleep consumption of CEM did not provide an additional advantage over consumption during the day in terms of prealbumin levels. While the literature suggests that presleep casein consumption may increase muscle protein synthesis [29], the reflection of this effect on biochemical parameters may require longer-term applications. However, the increase observed only in prealbumin levels in our study may indicate an early biochemical response to casein supplementation administered in the early post-bariatric surgery period.

This study has several limitations. The follow-up period was limited to three months, and the assessment of protein status relied on only a few biomarkers (prealbumin, albumin, and total protein), which may restrict the generalizability of the findings. The lack of statistically significant differences between groups may be partially attributed to the limited sample size, which may have rendered the study underpowered to detect subtle effects of protein timing. Future studies should incorporate longer follow-up periods, include inflammatory and sarcopenia-specific markers, and integrate objective physical activity monitoring in order to elucidate the long-term metabolic and functional effects of protein supplementation timing.

## 5. Conclusions

This study has several limitations. The follow-up period was limited to three months, and the assessment of protein status relied on only a few biomarkers (prealbumin, albumin, and total protein), which may restrict the generalizability of the findings. The lack of statistically significant differences between groups may be partially attributed to the limited sample size, which may have rendered the study underpowered to detect subtle effects of protein timing. It should also be considered that the timing of protein intake may not have had a significant effect on the measured variables. Moreover, the few differences observed between groups might have been influenced by the unequal amounts of protein provided, since Groups 1 and 2 received 75 g/day while Group 3 received 60 g/day. Future studies should incorporate longer follow-up periods, include inflammatory and sarcopenia-specific markers, and integrate objective physical activity monitoring in order to elucidate the long-term metabolic, functional, and healthy life effects of protein supplementation timing.

## Figures and Tables

**Table 1:** Comparison of anthropometric measurements of patients according to groups.

Time		Group 1		Group 2		Group 3		F/H	*p*
	Variables	Mean ± SD	Median (Min.–Max.)	Mean ± SD	Median (Min.–Max.)	Mean ± SD	Median (Min.–Max.)		
W4	EWL	16.4 ± 5.3	15.6 (9.2–27)	19.7 ± 11.2	18.6 (−6.8–36.6)	17.7 ± 10.3	16.3 (6.7–49.6)	1.788 ^b^	0.409
	EBMIL	20.6 ± 7.4	21.9 (9.9–35.4)	23.8 ± 13.1	23.3 (−7.3–47.1)	20.9 ± 11.4	18.0 (8.7–56.9)	2.590 ^b^	0.274
	FFM (kg)	60.5 ± 14.5	54.7 (43.1–88)	62.4 ± 12.7	57.7 (46–82.4)	62.5 ± 12.2	63.5 (44.2–81.2)	0.117 ^a^	0.890
	SMM (kg)	57.7 ± 14.3	51.9 (40.9–83.7)	59.3 ± 12.2	54.8 (43.7–78.4)	59.4 ± 11.7	60.3 (42–77.2)	0.085 ^a^	0.919
	BFM (kg)	48.0 ± 12.8	42.6 (35–76.1)	43.3 ± 12.9	39.1 (27.9–62.1)	45.0 ± 7.3	45.4 (32.2–61.2)	1.079 ^b^	0.583
	TBW (kg)	44.4 ± 12.5	37.2 (31.5–68.1)	45.5 ± 9.5	41.1 (33.5–61.2)	44.9 ± 9.2	40.6 (34.3–59)	0.543 ^b^	0.762
W8	EWL	28.0 ± 8.1	27.3 (18.3–47.7)	30.3 ± 14.1	32.2 (3.5–50.8)	31.7 ± 12.3	29.6 (13.8–56.3)	0.379 ^a^	0.687
	EBMIL	35.3 ± 12.2	34.3 (19.8–62.5)	36.4 ± 17.0	39.1 (4.6–67.5)	37.2 ± 12.5	37 (17.8–64.5)	0.070 ^a^	0.933
	FFM (kg)	58.6 ± 14.5	51.6 (40–82.5)	61.2 ± 11.8	56.5 (45.2–78.9)	59.4 ± 10.5	61.7 (44.2–76.2)	0.167 ^a^	0.847
	SMM (kg)	56.0 ± 13.7	51.0 (38–78.6)	58.2 ± 11.3	53.6 (42.9–75)	56.5 ± 9.9	58.6 (42–72.5)	0.139 ^a^	0.871
	BFM (kg)	42.8 ±12.6	39.0 (27–65.7)	39.0 ± 12.4	35 (24–59.7)	40.8 ± 8.1	41.4 (26–57.6)	0.429 ^a^	0.654
	TBW (kg)	42.6 ± 11.8	35.8 (30–62.8)	44.0 ± 8.8	39.6 (32.4–58.4)	42.3 ± 8.5	38.6 (30–56)	0.662 ^a^	0.718
W12	EWL	38.2 ± 8.4	38.1 (26–56.2)	40.9 ± 15.0	41.7 (12.6–67.6)	43.8 ± 15.2	46.2 (21.9–67.8)	0.654 ^a^	0.525
	EBMIL	47.9 ± 12.4	48.8 (28–73.7)	49.3 ± 18.6	49.7 (16.6–84.4)	51.7 ±16.6	49.7 (28.3–80.8)	0.219 ^a^	0.804
	FFM (kg)	57.8 ± 13.7	51.6 (39.2–80.7)	59.0 ± 11.2	54.9 (44–75.8)	58.0 ± 10.2	58.5 (42.3–76.7)	0.044 ^a^	0.957
	SMM (kg)	54.9 ±13.1	49.0 (37–76.7)	55.9 ± 10.8	52.1 (41–72.1)	53.8 ± 9.5	53.6 (40.1–72.9)	0.130 ^a^	0.879
	BFM (kg)	38.1 ±10.0	34.3 (24.1–55.2)	35.6 ± 11.9	32.0 (17.9–53.5)	35.8 ± 8.6	36.0 (24–49.7)	0.272 ^a^	0.763
	TBW (kg)	40.9 ± 11.2	35.1 (30.1–60.6)	39.7 ± 7.7	37.0 (31.9–55.8)	40.9 ± 8.0	35.5 (32.5–55.2)	0.606 ^b^	0

EWL: excess weight loss; EBMIL: excess body mass index loss; FFM: fat-free mass; BFM: body fat mass; SMM: skeletal muscle mass; TBW: total body water; W: week. a: one way ANOVA test, b: Kruskal Wallis test.

**Table 2:** Comparison of handgrip strength and 30 s sit-to-stand test measurements according to groups.

Variables	Time	Group 1		Group 2		Group 3		F/H	*p*
		Mean ± SD	Median (Min.–Max.)	Mean ± SD	Median (Min.–Max.)	Mean ± SD	Median (Min.–Max.)		
RH	Preop	29.2 ±8.2	27.7 (18.9–45.6)	34.5 ± 12.4	32.3 (18.3–51.5)	32.3 ± 12.8	28.3 (15.2–56.7)	0.958 ^b^	0.619
	W4	31.4 ± 9.1	28.3 (20–48.7)	36.8 ± 12.8	36.7 (18–56.1)	32.6 ± 12.5	29.0 (15.1–53.1)	0.905 ^a^	0.412
	W8	31.8 ± 9.3	30.0 (21–53.1)	36.9 ±12.4	35.4 (19–57)	32.2 ± 12.6	29.0 (13.6–54)	0.897 ^a^	0.416
	W12	33.7 ± 10.4	30.5 (22.9–55)	36.7 ± 11.8	34.0 (20–58)	33.5 ±11.3	29.9 (15.3–54)	0.550 ^b^	0.759
LH	Preop	29.6 ± 9.1	27.1 (19.1–45.1)	33.3 ± 12.2	29.7 (16.9–51.4)	31.1 ± 12.0	27.1 (12–51.4)	0.418 ^a^	0.661
	W4	31.2 ± 9.0	28.7 (20.4–45.3)	35.4 ± 13.1	31.4 (17–55.1)	32.9 ± 12.3	28.0 (14.7–52.1)	0.496 ^a^	0.613
	W8	32.0 ± 9.3	29.0 (20–50.3)	36.0 ± 12.8	31.9 (18–56)	32.5 ± 11.5	29.0 (16–53)	0.555 ^a^	0.578
	W12	32.8 ± 10.4	29.2 (20.2–52)	35.6 ± 12.2	31.5 (20–57)	32.5 ± 11.3	28.0 (16.2–55)	0.342 ^a^	0.713
STS	Preop	10.5 ± 1.9	11.0 (7–13)	11.4 ± 1.8	12.0 (9–15)	11.1 ± 3.3	10.0 (7–19)	1.073 ^b^	0.585
	W4	11.6 ± 1.9	12.0 (8–14)	12.9 ± 2.6	13.0 (10–20)	12.3 ± 3.6	11.0 (8–23)	1.581 ^b^	0.454
	W8	12.3 ± 1.8	13.0 (9–15)	13.7 ± 2.7	13.0 (10–21)	12.9 ± 3.7	12.0 (8–24)	1.992 ^b^	0.369
	W12	13.7 ± 2.2	14.0 (9–17)	14.4 ± 2.9	14.0 (10–22)	13.5 ± 4.1	12.0 (8–25)	1.725 ^b^	0.422

RH: right hand; LH: left hand; STS: sit-to-stand test; W: week. a: one way ANOVA test, b: Kruskal Wallis test.

**Table 3:** Comparison of biochemical parameter measurements according to groups.

Variables		Group 1		Group 2		Group 3		F/H	*p*
		Mean ± SD	Median (Min.–Max.)	Mean ± SD	Median (Min.–Max.)	Mean ± SD	Median (Min.–Max.)		
Preop	Prealbumin	0.3 ± 0	0.3 (0.2–0.3)	0.2 ± 0.1	0.2 (0.1–0.3)	0.3 ± 0.0	0.3 (0.2–0.3)	4.651 ^a^	0.015 *
	Albumin	4.4 ± 0.3	4.3 (4.0–4.7)	4.8 ± 0.3	4.7 (4.5–5.4)	4.5 ± 0.3	4.5 (4.1–5.0)	10.416 ^b^	0.005 *
	Total Protein	7.4 ± 0.4	7.2 (6.5–8.1)	7.6 ± 0.3	7.6 (7.0–8.5)	7.4 ± 0.4	7.4 (6.7–8.0)	2.485 ^b^	0.289
W4	Prealbumin	0.2 ± 0	0.3 (0.1–0.3)	0.2 ± 0.1	0.2 (0.1–0.5)	0.2 ± 0.0	0.2 (0.1–0.3)	12,434 ^b^	0.002 *
	Albumin	4.4 ± 0.3	4.5 (3.9–4.9)	4.6 ± 0.2	4.6 (4.0–5.0)	4.5 ±0.4	4.5 (3.9–5.3)	0.922 ^a^	0.406
	Total Protein	7.1 ± 0.4	7.1 (6.3–7.9)	7.2 ± 0.3	7.1 (6.8–7.8)	7.1 ±0.5	7.4 (6.0–7.7)	0.617 ^a^	0.544
W8	Prealbumin	0.2 ± 0	0.3 (0.2–0.3)	0.2 ± 0.0	0.2 (0.2–0.3)	0.2 ±0.0	0.2 (0.2–0.3)	13.713 ^b^	0.001 *
	Albumin	4.5 ± 0.4	4.5 (3.7–4.9)	4.6 ± 0.2	4.5 (4.2–5.0)	4.4 ± 0.3	4.5 (4.0–5.0)	3.659 ^b^	0.160
	Total Protein	7.1 ± 0.4	7.0 (6.5–8.0)	7.2 ± 0.3	7.0 (6.7–7.5)	7.0 ±0.4	7.0 (6.5–7.5)	1.360 ^b^	0.507
W12	Prealbumin	0.3 ± 0.1	0.2 (0.2–0.5)	0.2 ±0.0	0.2 (0.2–0.3)	0.2 ±0.0	0.2 (0.1–0.2)	16,699 ^b^	*p* < 0.001
	Albumin	4.5 ± 0.3	4.4 (4.0–4.9)	4.5 ±0.2	4.5 (4.0–4.7)	4.4 ±0.2	4.4 (4.0–4.8)	1.742 ^b^	0.419
	Total Protein	7.2 ± 0.4	7.1 (6.5–8.3)	10.9 ± 15.0	7.0 (6.5–65.0)	7.0 ±0.3	7.0 (6.4–7.4)	2.307 ^b^	0.316

W: week. a: one way ANOVA test, b: Kruskal Wallis test, *: *p* < 0.05.

## Data Availability

The data obtained in this study are available from the corresponding author upon request.

## Supplementary Materials

The following supporting information can be downloaded at: https://www.mdpi.com/article/10.3390/nu17172750/s1. Table S1: comparison of preoperative anthropometric measurements across groups; Table S2: comparison of handgrip strength and 30 s sit-to-stand test results over time.

## Abbreviations

The following abbreviations are utilized in this manuscript:

SG	Sleeve gastrectomy
CEM	Casein-enriched milk
FFM	Fat-free mass
BFM	Body fat mass
SMM	Skeletal muscle mass
TBW	Total body water
BMI	Body mass index
EWL	Excess weight loss
EBMIL	Excess body mass index loss
STS	Sit-to-stand test
W	Week
RH	Right hand
LH	Left hand
