# Supplementary material for: Presleep vs. Daytime Consumption of Casein-Enriched Milk: Effects on Muscle Function and Metabolic Health After Sleeve Gastrectomy
Source: Nutrients. 2025 Aug 25;17(17):2750. doi: 10.3390/nu17172750 (PMC12430192; doi:10.3390/nu17172750)
Supplement: Supplementary file 1 [file nutrients-17-02750-s001.zip › nutrients-3810052-supplementary.pdf]

Supplementary Tables

Table S1. Comparison of preoperative anthropometric measurements across groups.

	Group 1		Group 2		Group 3			
**Variables**	**Mean ± SD**	**Median (Min.-Max.)**	**Mean ± SD**	**Median (Min.-Max.)**	**Median ± SD**	**Median (Min.-Max.)**	**F/H**	**p**
BMI (kg/m²)	42.3 ± 6.1	39.8 (35.7-54.3)	41.0 ± 5.2	39.6 (35.4-50.6)	41.0 ± 3.1	39.9 (36.9-47.1)	0.369[a]	0.694
FFM (kg)	64.4 ± 15.1	59.1 (46.7-91.4)	66.7 ± 12.9	61.1 (48.3-84.6)	66.3 ± 13.4	67.6 (45.8-86.9)	0.120[a]	0.887
FFM (%)	54.7 ± 3.1	54.8 (47.7-61.7)	58.0 ± 7.9	58.0 (44.6-69.9)	56.2 ± 7.3	58.1 (40.1-67.9)	0.895[a]	0.382
SMM (kg)	61.2 ± 14.4	56.1 (44.3-87)	63.0 ± 12.9	58.0 (43.8-80.5)	63.0 ± 12.7	64.2 (43.5-82.7)	0.092[a]	0.912
SMM (%)	51.9 ± 2.9	52.0 (45.3-58.4)	54.7 ± 8.0	55.0 (42.3-66.4)	53.4 ± 7.0	55.3 (38.1-64.6)	0.711[a]	0.497
FM (kg)	53.6 ± 14.0	49.4 (38.2-84.4)	48.8 ± 13.5	44.3 (34.9-70.1)	49.6 ± 8.0	48.8 (36-66.7)	1.465[b]	0.481
FM (%)	45.3 ± 3.1	45.2 (38.3-52.3)	42.0 ± 7.9	42.0 (30.1-55.4)	42.4 ± 5.9	40.9 (32.1-52.2)	1.398[a]	0.258
TBW (kg)	47.2 ± 13.5	40.2 (33.9-73)	48.7 ± 9.9	43.4 (35.6-63.5)	47.3 ±10.4	42.2 (34.8-64.7)	0.729[b]	0.695
TBW (%)	39.7 ± 3.3	39.5 (33.2-45.2)	42.3 ± 6.0	42.7 (33.1-49.6)	40.2 ± 5.8	37.8 (26.9-49.3)	1.061[a]	0.355

BMI: body mass index loss; FFM: fat free mass; SMM: skeletal muscle mass; TBW: total body water.

Table S2. Comparison of hand grip strength and 30-second sit-to-stand test results over time

		Baseline		W4		W8		W8			
	**Variables**	**Mean ± SD**	**Median (Min.-Max.)**	**Mean ± SD**	**Median (Min.-Max.)**	**Mean ± SD**	**Median (Min.-Max.)**	**Mean ± SD**	**Median (Min.-Max.)**	**Ki-kare**	**p**
Group 1	RH	29.2 ±8.2	27.7 (18.9-45.6)	31.4 ± 9.1	28.3 (20-48.7)	31.8 ± 9.3	30.0 (21-53.1)	33.7 ± 10.4	30.5 (22.9-55)	22.212[b]	p<0.001
	LH	29.6 ± 9.1	27.1 (19.1-45.1)	31.2 ± 9.0	28.7 (20.4-45.3)	32.0 ± 9.3	29.0 (20-50.3)	32.8 ± 10.4	29.2 (20.2-52)	15.776[b]	0.001*
	STS	10.5 ± 1.9	11.0 (7-13)	11.6 ± 1.9	12.0 (8-14)	12.3 ± 1.8	13.0 (9-15)	13.7 ± 2.2	14.0 (9-17)	35.63[b]	p<0.001*
Group 2	RH	34.5 ± 12.4	32.3 (18.3-51.5)	36.8 ± 12.8	36.7 (18-56.1)	36.9 ±12.4	35.4 (19-57)	36.7 ± 11.8	34.0 (20-58)	13.399[b]	0.004*
	LH	33.3 ± 12.2	29.7 (16.9-51.4)	35.4 ± 13.1	31.4 (17-55.1)	36.0 ± 12.8	31.9 (18-56)	35.6 ± 12.2	31.5 (20-57)	8.301[b]	0.040*
	STS	11.4 ± 1.8	12.0 (9-15)	12.9 ± 2.6	13.0 (10-20)	13.7 ± 2.7	13.0 (10-21)	14.4 ± 2.9	14.0 (10-22)	41.935[b]	p<0.001
Group 3	RH	32.3 ± 12.8	28.3 (15.2-56.7)	32.6 ± 12.5	29.0 (15.1-53.1)	32.2 ± 12.6	29.0 (13.6-54)	33.5 ±11.3	29.9 (15.3-54)	2.83[b]	0.419
	LH	31.1 ± 12.0	27.1 (12-51.4)	32.9 ± 12.3	28.0 (14.7-52.1)	32.5 ± 11.5	29.0 (16-53)	32.5 ± 11.3	28.0 (16.2-55)	3.166[b]	0.367
	STS	11.1 ± 3.3	10.0 (7-19)	12.3 ± 3.6	11.0 (8-23)	12.9 ± 3.7	12.0 (8-24)	13.5 ± 4.1	12.0 (8-25)	35.723[b]	p<0.001

RH: right hand; LH: left hand; STS: sit to stand test.
